# Draft Genome Sequence of Clostridium sp. Strain chh4-2 Isolated from Human Feces

**DOI:** 10.1128/genomeA.00070-18

**Published:** 2018-02-22

**Authors:** Chien-Hsun Huang, Jong-Shian Liou, Chun-Lin Wang, Lina Huang

**Affiliations:** a Bioresource Collection and Research Center, Food Industry Research and Development Institute, Hsinchu, Taiwan, Republic of China

## Abstract

Here, we report the draft genome sequence of a Clostridium sp. strain isolated from a fecal sample of a 34-year-old adult male in Taiwan. This strain may represent a new bacterium, as suggested by a comparison based on whole-genome sequencing. The genome assembly comprised 6,089,737 bp, with a 45.63% G+C content.

## GENOME ANNOUNCEMENT

Culturomics technology is a powerful tool for capturing new species in the human gut, one that can be used to overcome depth-biased problems with metagenomic approaches (1). We isolated strain chh4-2 from a fecal sample of a 34-year-old adult male in Taiwan during a study aimed at isolating new bacterial species present in the human gut using a culture-based method. This method included serial dilution and plating combined with matrix-assisted laser desorption ionization–time of flight (MALDI-TOF) mass spectrometry and 16S rRNA gene sequencing for strain dereplication and species identification. The 16S rRNA gene sequence analysis revealed that strain chh4-2 was related to Clostridium saccharolyticum NRC 2533^T^ (95.38 %), indicating that this isolate may represent a new species.

Clostridium sp. chh4-2 is a rod-shaped, strictly anaerobic, spore-forming bacterium. It grows on modified Gifu anaerobic medium agar (Nissui Pharmaceutical Co., Tokyo, Japan) within 48 h at 37°C under anaerobic conditions. Genomic DNA was extracted using an EasyPrep HY genomic DNA extraction kit (Tools, Taiwan) following the manufacturer’s protocol. The draft genome was sequenced from an Illumina paired-end library with an average insert size of 350 bp, using an Illumina HiSeq 4000 instrument with the paired-end 150-bp strategy at the Beijing Novogene Bioinformatics Technology Co., Ltd. (Beijing, China). Sequencing of strain chh4-2 generated 1,330 Mb of data. After adapter filtering and quality trimming, *de novo* assembly of the reads was performed using SOAPdenovo (2), which resulted in 61 scaffolds with an *N*_50_ value of 179,767 bp. The draft genome contains 6,089,377 bp, with a GC content of 45.63%, and was annotated using the NCBI Prokaryotic Genome Automatic Annotation Pipeline (PGAAP) (3) to obtain 5,343 predicted genes, 13 rRNAs, 64 tRNAs, and 4 noncoding RNAs. Annotation of the protein-coding genes with Rapid Annotations using Subsystems Technology (RAST) (4) assigned 25% of them to functional categories of SEED subsystems, while analysis with AntiSmash version 4.0 (5) predicted 12 biosynthetic clusters, which includes xantholipin and exopolysaccharide biosynthetic gene clusters.

In addition, the average nucleotide identity (ANI) value was estimated using orthologous ANI (OrthoANI) (6), and *in silico* DNA-DNA hybridization (isDDH) was performed using the genome-to-genome distance calculator (GGDC) with the recommended Formula 2 (7). The ANI and isDDH values between the novel strain and the C. saccharolyticum type strain were 71.1% and 21.7%, respectively, which were clearly lower than the generally accepted cutoff thresholds of ∼95.5% and 70.0% for delineating prokaryotic species, thus confirming that strain chh4-2 represents a novel species.

### Accession number(s).

This whole-genome shotgun project has been deposited at DDBJ/ENA/GenBank under the accession number PKUU00000000 (BioProject number PRJNA428016). The version described in this paper is the first version.
